# Editorial: Public Health Genomics

**DOI:** 10.3389/fpubh.2019.00142

**Published:** 2019-06-06

**Authors:** Paul Lacaze, Gareth Baynam

**Affiliations:** ^1^Public Health Genomics, Department of Epidemiology and Preventive Medicine, Monash University, Melbourne, VIC, Australia; ^2^Western Australian Register of Developmental Anomalies, King Edward Memorial Hospital, Perth, WA, Australia; ^3^Genetic Services of Western Australia, King Edward Memorial Hospital, Perth, WA, Australia; ^4^Office of Population Health Genomics, Public and Aboriginal Health Division, Perth, WA, Australia; ^5^Division of Paediatrics, Faculty of Health and Medical Sciences, University of Western Australia, Perth, WA, Australia; ^6^Telethon Kids Institute, University of Western Australia, Perth, WA, Australia

**Keywords:** genomics, public health, precision medicine, precision public health, ELSI

The term “Public Health Genomics” encompasses the many different areas where genomic information is used in public and population health. Primarily, this includes the use of human genotype in the prevention or treatment of disease. However, it also encompasses the use of pathogen genomics for outbreak monitoring, molecular profiling of tumor tissue for targeted therapies, and other areas.

Public Health Genomics (PHG) research also addresses regulatory and policy issues, related to the use of genetic information in society. In addition, it encompasses Ethical, Legal, and Social Issues (ELSI) raised by the growth and expansion of genomics. For example, newly emerging areas such as direct-to-consumer genetic testing via the internet and the use genetic information forensically for crime-solving purposes (1) are fast becoming PHG issues.

This research topic aims to provide an overview and introduction to the field of PHG. Articles have used language and addressed topics that we consider to be accessible for a general audience, including public health researchers not working in the field of genomics. Our intention is to introduce some of the key developments and challenges of the field, during a critical growth period.

Many articles focus on the Australian healthcare system and related policy, where progress has been made—including specific efforts underway to implement genomics into routine healthcare, address ELSI issues, and develop required PHG policy.

The series begins with Perspective articles on the history and evolution of PHG as a field. These provide an overview of some of the key issues and emerging trends, and how the field is currently poised. Molster et al. describe a range of activities that illustrate how genomics can be incorporated into public health practice. They present the evolution of public health genomics into the new era of “precision public health,” which put simply is using the best available data to target more effectively and efficiently interventions of all kinds to those most in need (2, 3). Bilkey et al. discuss the potential impacts of precision medicine on public health policy and decision-making, with particular focus on patients living with rare diseases and rare cancers. They present precision public health as the bridge between precision medicine and public health. Burns et al. explore priority-setting for sustainable implementation of genomic testing into healthcare within the strategic priority areas of the Australian National Health Genomics Policy Framework. The priority areas include services, data, workforce, finances, and person-centered care. They argue that for full effectiveness resources should not be allocated genomic testing alone, but should cover all these priority areas.

The research topic then focuses on ELSI, including a review of issues across the lifespan of genomic testing—from newborn bloodspot screening, to adult predisposition testing, to reproductive carrier screening, to molecular autopsy (Bilkey et al.).

Articles then focus on a particularly pressing and topical issue in PHG: the use of genetic test results in insurance underwriting. Tiller et al. provide a detailed account of the ethical and regulatory situation in Australia, amidst the ongoing use of genetic test results in life insurance underwriting. Concerns persist around industry self-regulation and lack of government oversight on this issue in Australia (4). In a separate study, Tiller et al. present original research, collecting quantitative survey data from genetic health professionals on workforce trends, practices, and knowledge around genetic testing and life insurance regulation in Australia. They report considerable variation amongst survey respondents (genetics professionals), genetic health services, and geographic locations regarding understanding and communication of current regulation. The evolving US regulatory landscape around employer use of genetic information is then considered by Bilkey et al., including implications for Australia and other jurisdictions.

Beyond the issue of insurance, Tiller and Lacaze also consider the difficulty of regulating internet-based genetic testing, in a rapidly evolving landscape. It is now estimated that over 26 million people have taken at-home ancestry tests—an unprecedented level of testing. This is mostly unregulated, raising several issues, including the practice of consumers imputing raw genotyping data from ancestry companies using third-party online tools to generate medical risk estimates of questionable quality (5). This can lead to confusion, unexpected findings, and an increased burden on local genetic health services (6).

Ryan et al. address the complex issue of dementia prevention for the aging population. Here, considerable biological and phenotypic heterogeneity in dementia make biomarker development challenging. Genomic and other ‘omic approaches provide opportunities for novel biomarker classes (7), however far more research and development is still required.

Finally, Nunn et al. conduct a scoping review of public involvement in global genomics research. This is the first study of its kind to consider the degree of public involvement occurring in prominent human genomics projects around the world. The study suggests current levels of public involvement need to be improved, as the level of genomic research and testing in society approaches population scale (8).

Together, the Research Topic provides a broad and diverse overview of a field that is rapidly evolving. Articles are timely and address real-world issues. Genomics has the potential to improve the way we deliver healthcare and precision public health in the future. However, the many ethical, regulatory, and scientific challenges must be carefully addressed in coming years, if these benefits are to be realized.

## Author Contributions

PL and GB edited the Research Topic and wrote the manuscript.

### Conflict of Interest Statement

The authors declare that the research was conducted in the absence of any commercial or financial relationships that could be construed as a potential conflict of interest.
